# *PRRT2* gene variant in a child with dysmorphic features, congenital microcephaly, and severe epileptic seizures: genotype-phenotype correlation?

**DOI:** 10.1186/s13052-019-0755-2

**Published:** 2019-12-04

**Authors:** Piero Pavone, Giovanni. Corsello, Sung Yoon Cho, Xena Giada Pappalardo, Martino Ruggieri, Simona Domenica Marino, Dong Kyu Jin, Silvia Marino, Raffaele Falsaperla

**Affiliations:** 1 Department of Pediatrics, University-Hospital “Policlinico-Vittorio Emanuele”, University of Catania, Via Santa Sofia 78, 95124 Catania, Italy; 2 Institute of Pediatrics, University of Palermo, Palermo, Italy; 3 Department of Pediatrics, Samsung Medical Center, Sungkyunkwan University School of Medicine, Seoul, South Korea; 4 National Council of Research, CNR, Institute for Research and for Biomedicine Innovation (IRIB) unit of Catania, Catania, Italy; 5 Department of Clinical and Experimental Medicine, Section of Pediatrics and Child Neuropsychiatry, University of Catania, Catania, Italy

**Keywords:** *PRRT2* mutation, Dysmorphic features, Microcephaly, Epileptic encephalopathy

## Abstract

**Background:**

Mutations in Proline-rich Transmembrane Protein 2 (*PRRT2*) have been primarily associated with individuals presenting with infantile epilepsy, including benign familial infantile epilepsy, benign infantile epilepsy, and benign myoclonus of early infancy, and/or with dyskinetic paroxysms such as paroxysmal kinesigenic dyskinesia, paroxysmal non-kinesigenic dyskinesia, and exercise-induced dyskinesia. However, the clinical manifestations of this disorder vary widely. *PRRT2* encodes a protein expressed in the central nervous system that is mainly localized in the pre-synaptic neurons and is involved in the modulation of synaptic neurotransmitter release. The anomalous function of this gene has been proposed to cause dysregulation of neuronal excitability and cerebral disorders.

**Case presentation:**

We hereby report on a young child followed-up for three years who presents with a spectrum of clinical manifestations such as congenital microcephaly, dysmorphic features, severe intellectual disability, and drug-resistant epileptic encephalopathy in association with a synonymous variant in *PRRT2* gene (c.501C > T; p.Thr167Ile) of unknown clinical significance variant (VUS) revealed by diagnostic exome sequencing.

**Conclusion:**

Several hypotheses have been advanced on the specific role that *PRRT2* gene mutations play to cause the clinical features of affected patients. To our knowledge, the severe phenotype seen in this case has never been reported in association with any clinically actionable variant, as the missense substitution detected in *PRRT2* gene. Intriguingly, the same mutation was reported in the healthy father: the action of modifying factors in the affected child may be hypothesized. The report of similar observations could extend the spectrum of clinical manifestations linked to this mutation.

## Introduction

Reports on individuals with Proline-rich Transmembrane Protein 2 (*PRRT2*) mutations have varied greatly in the clinical expressions, but most involve benign epilepsies and/or dyskinetic paroxysms [1–7] which may present in association [8] or within the same family [9]. At an early age, the *PRRT2* mutations have been associated to benign familial infantile epilepsy, benign infantile epilepsy, and benign myoclonus of early infancy [2, 4, 5]. The clinical features of infants with benign infantile epilepsy are relativity typical, with seizures beginning in the first days or months of life and tending to disappear very early, usually within the first few years [10]. In these infants, their developmental stages and physical examination are within the normal range. The *PRRT2* mutations have also been linked to paroxysmal dyskinesia, a group of disorders in which the affected children show episodic, sudden abnormal movements that are not associated with loss of consciousness. These disorders include paroxysmal kinesigenic dyskinesia (PKD), triggered by voluntary or involuntary movements; paroxysmal non-kinesigenic dyskinesia, which is not triggered by voluntary movements; and paroxysmal exercise-induced dyskinesia, triggered by repetitive motions and physical exercise [2, 3, 6, 7]. The *PRRT2* mutations have also been reported in familial cases of hemiplegic migraine, in children with benign paroxysmal torticollis, and in individuals with progressive and stable ataxia [1, 2, 11, 12]. Only rarely have been reported patients with *PRRT2* mutations presenting with severe neurological impairment such as focal seizures and epileptic spasms [13], severe epileptic seizures, cognitive disabilities, or complex malformations [14–16]. However, the clinical manifestations may vary widely as reported by Igarashi et al. [13] who described a girl with Down syndrome (47, XY + 21) who exhibited focal seizures and epileptic spasms during infancy. Her younger brother had focal seizures at five months and a normal development with normal interictal electroencephalogram. The father had infantile spasms and benign PKD typical of *PRRT2*-related manifestations. In the three patients, a mutation analysis of the *PRRT2* gene disclosed a 841 T > C mutation. We report on a young child with congenital microcephaly, facial dysmorphisms, and severe epileptic encephalopathy. In this study, a comparative genomic microarray analysis (aCGH) and a clinical exome sequencing were carried out for the family trio (both parents and their affected child sequenced simultaneously) to effectively detect de novo and compound heterozygous variants causing epileptic encephalopathy (EE), motor and cognitive delay and seizure unresponsive to treatment. Unsurprisingly, no rearrangements were found in aCGH test and the gene panel sequencing identified however, a heterozygous synonymous VUS in the gene *PRRT2* (c.501 C > T; p.Thr 167 =), inherited from the healthy father.

## Case report

The boy was first referred to our consultation at age of 2 months for a diagnostic work-up due to a neurological assessment and epileptic seizures. The father is Italian, and the mother is originally from Cuba but is now a nationalized Italian citizen. The father was 31-years old and the mother 27 at the time of gestation, during which the mother felt fetal movements normally. The pregnancy was uneventful apart from some episodes of vomiting happened in the first month of pregnancy. The mother denied having had infectious disease or having used drugs or alcohol during the pregnancy. At the fetal age of 6 months, an intrauterine ultrasound disclosed a head circumference in the lower range, but the parents refused to interrupt the pregnancy. Just before the delivery, the mother was in a comatose state that lasted a few hours, and a cesarean section was rapidly performed. The child was born after 38 weeks’ gestation with a birth weight of 2800 g, length of 48 cm, and head circumference of 31 cm. The Apgar scores at 1 and 5 min were 5 and 7, respectively. The child showed severe respiratory distress at birth, and was immediately admitted to the neonatal intensive care unit where he was endotracheally intubated for 7 days. Facial dysmorphisms were noted from the first hours of life and the child exhibited abnormal clonic movements of the upper and lower limbs, which were treated with intramuscular phenobarbital at 10 mg/kg/day with poor results. He was discharged from the hospital 1 month after admission.

**Fig. 1:**
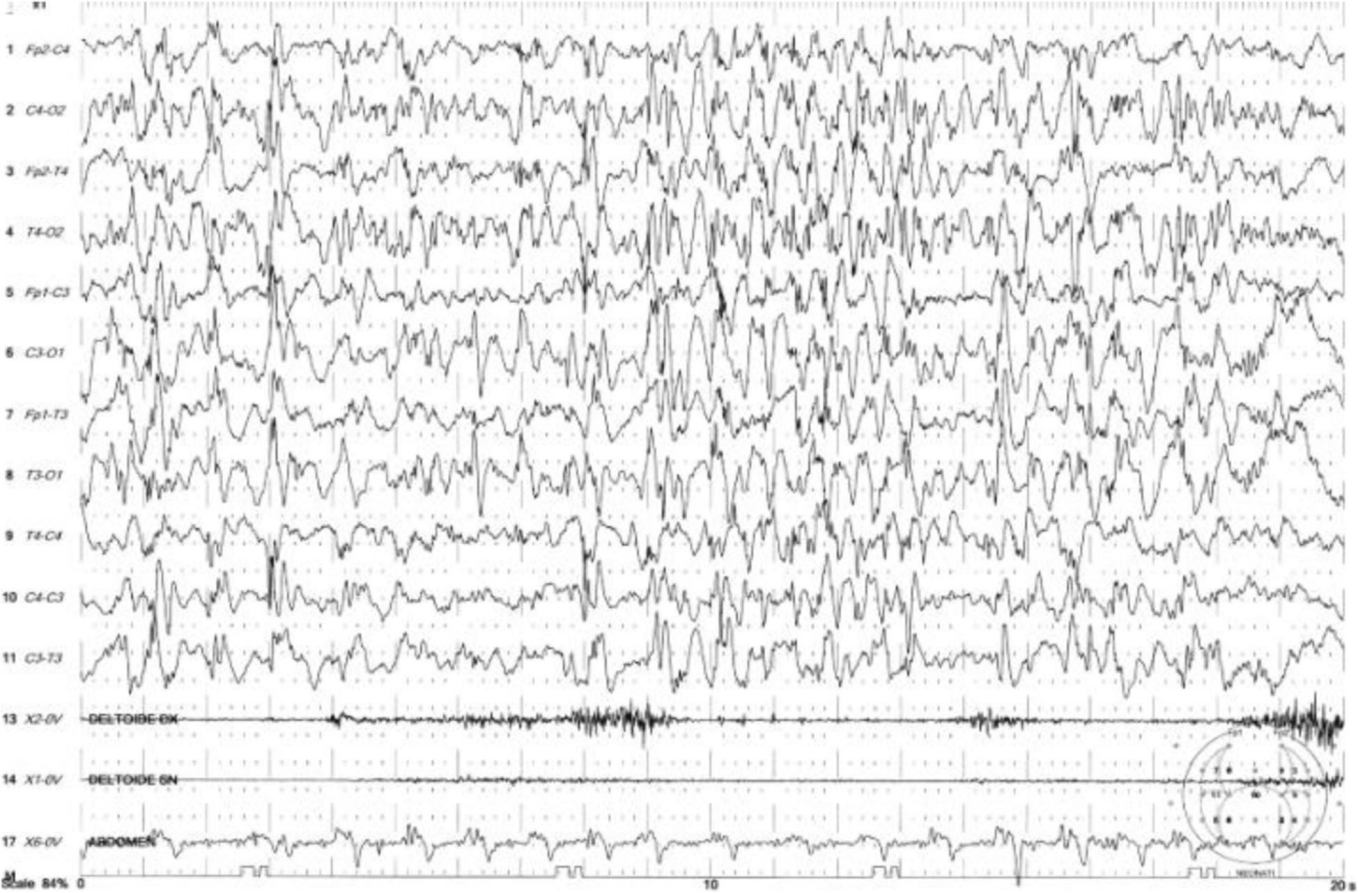
Intercritical video-EEG performed at 3 months: showing slow spikes in the right occipital region with dys-regulated background

**Fig. 2:**
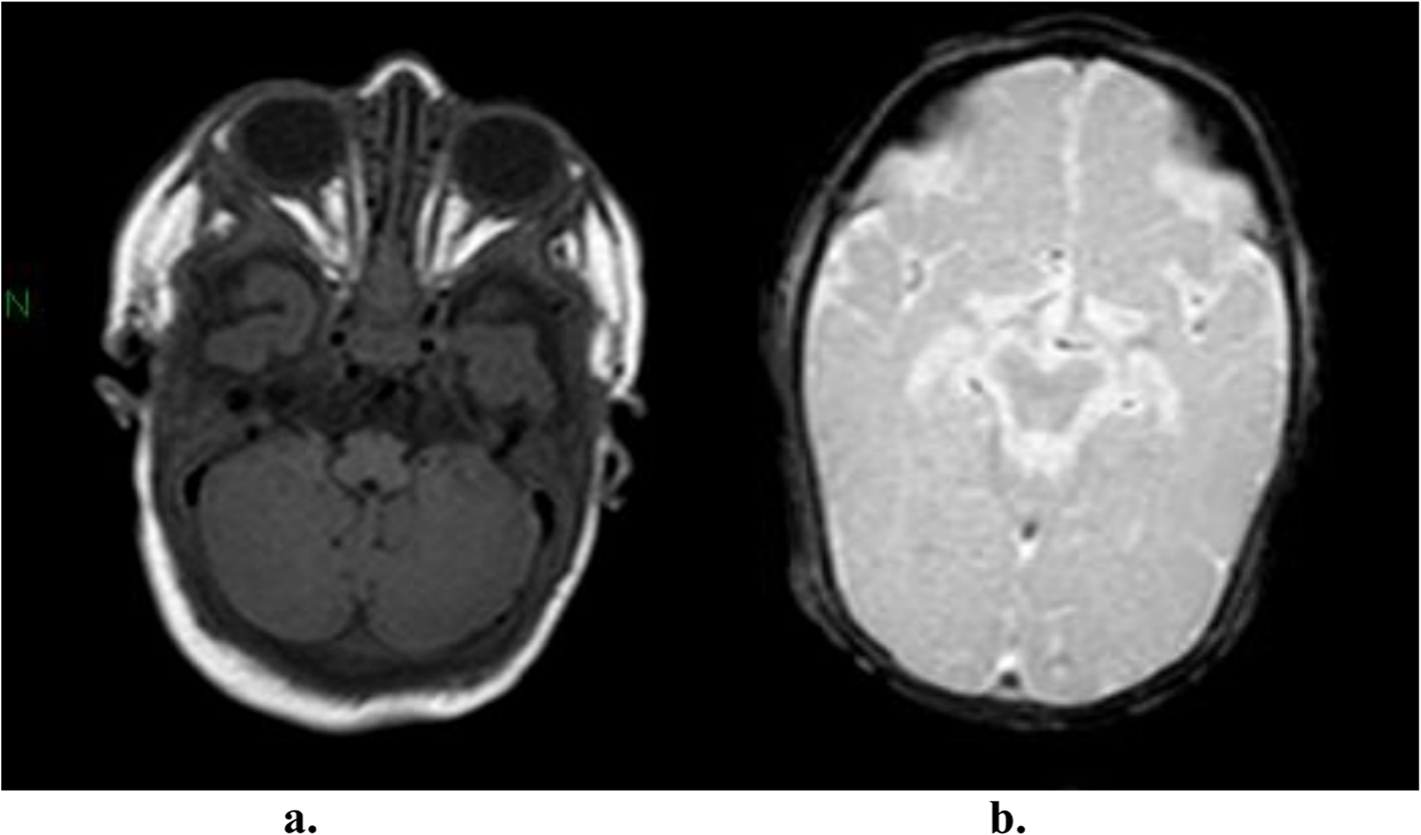
**a**-**b** Brain MRI performed at 3 months: **a** T1 coronal sequence showing simplified cortical structures (shallow sulci); **b** T2 coronal sequence showing micro cavitation localized in the pre and post rolandic subcortical white matter areas

The boy first came to our observation at the age of 2 months with a weight of 3200 g, length of 53 cm, and head circumference of 34 cm (< 3 SD). During the hospitalization, the infant showed severe generalized hypotonia and abnormal movements in clusters, mainly of the migrant myoclonic type and localized to the upper limbs. Episodes of horizontal gaze nystagmus were also noted. There was a lack of eye contact, and the patellar reflexes were poorly elicited. At this time, the EEG showed slow spikes in the right occipital region with dysregulated background activity, and a brain ultrasound showed severe structural damage. An electrocardiogram, thorax X-ray, and abdominal ultrasound were normal. Hemogram, electrolytes, urinalysis, TORCH screen, and inflammatory marker test gave normal results. Urinary organic acid and plasma amino acid analyses were also normal. Deglutition was impaired with severe dysphagia, and the deglutition reflex was absent, requiring the infant be to fed by nasogastric tube. The phenobarbital was increased to 7.5 mg twice a day, but the seizures persisted. At a new admission at 3 months of age, the movements were mainly dystonic and presenting during sleep. Hypotonia was severe and generalized. A video-EEG displayed multifocal, paroxysmal spike and wave activity that did not correlate with the dystonic movement (Fig. 1). Adrenocorticotropic hormone (ACTH) treatment was administered first for 2 weeks, and then with valproate as an add-on, with poor response. The brain MRI showed simplified cortical structures (shallow sulci) with microcavitation localized primarily in the pre- and post-rolandic subcortical white matter areas (Fig. 2 a, b). Otoacoustic emission, impedentiometric, and visual and auditory evoked potential analyses were normal. In an attempt to reduce epileptic seizures anticonvulsant treatment with levetiracetam, valproate, and topiramate, in isolation or in add-on, and a ketogenic diet were carried out, but with poor results. The child was followed-up over serial hospitalizations every two to three months due to epileptic seizures of various types, usually tonic rather than myoclonic, and spasms in flexion with episodes of convulsive status epilepticus. During the subsequent months, the child continued to show drug-resistant seizures, persistent electroencephalographic abnormalities, and severe developmental delay.

**Fig. 3:**
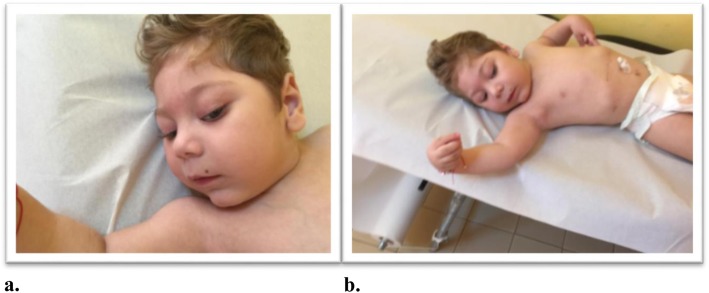
**a**-**b**. At the age of two-and-a-half years, the child notes: **a** a small and receding forehead, sparse eyebrows, epicanthus, upslanting palpebral fissures, full cheeks, a wide nasal bridge, rounded nasal tip, large nares, **b** The wrist and fingers were flexed with the hands partially closed

**Fig. 4:**
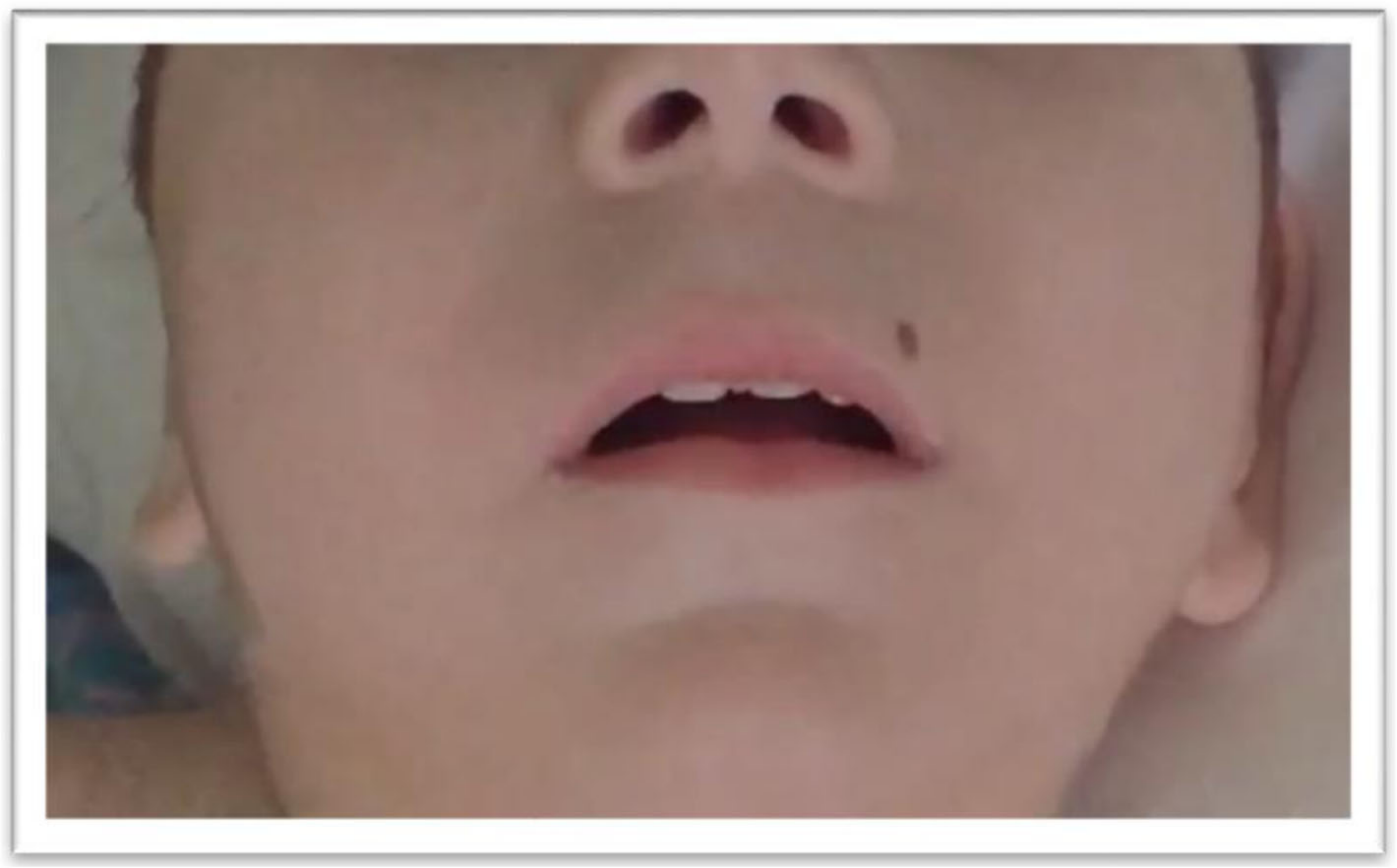
At 3 years, to note: long philtrum, small mouth, thin lips and a horizontal furrow under the lower lips, retrognathia and a small nevus localized on the upper lip

**Fig. 5:**
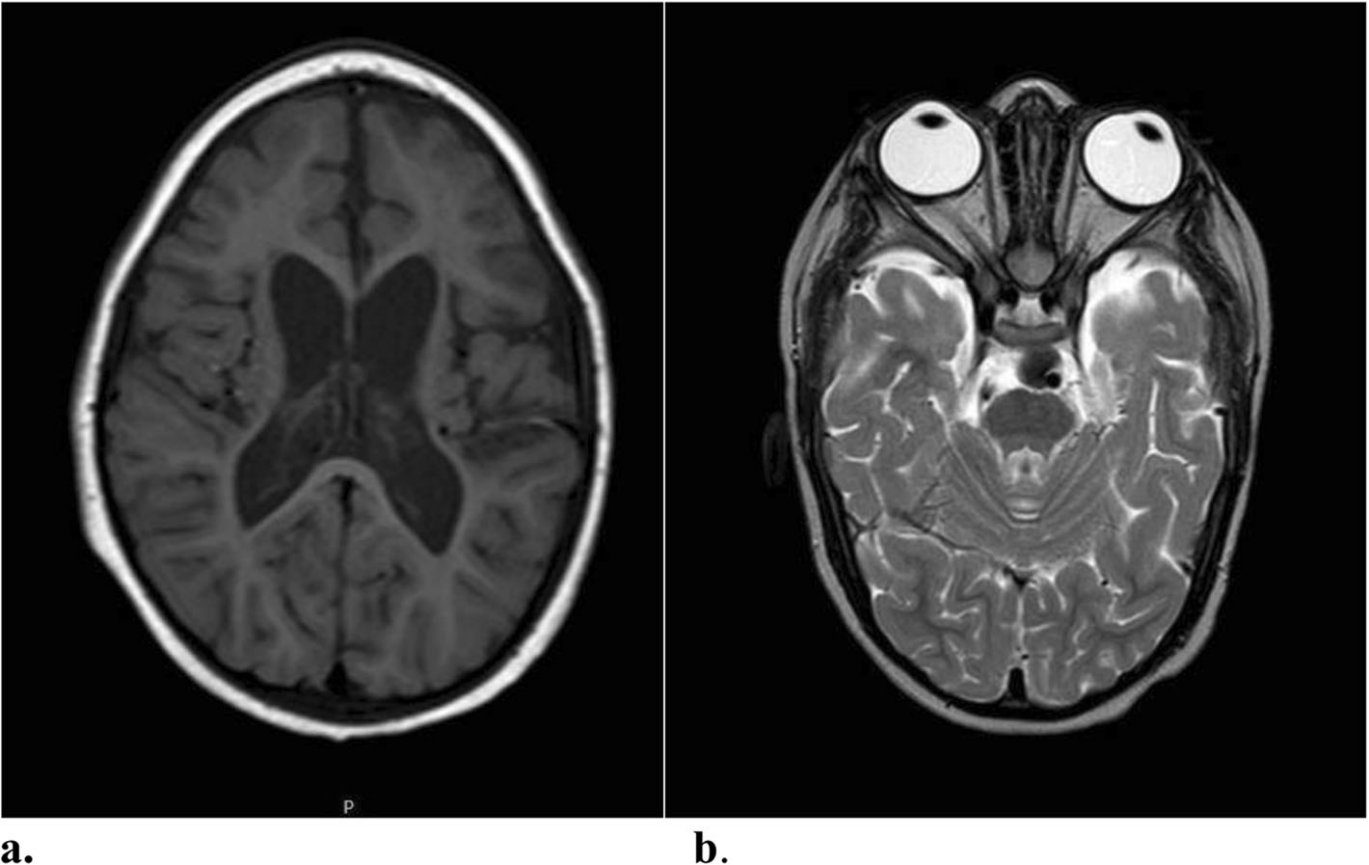
**a**-**b**: Brain MRI performed at 3 years: **a**) Axial T1-weighted image and **b**) Axial T2-weighted image: moderate enlargement of both lateral ventricles and subarachnoid space of bilateral temporal pole with volume reduction of the supratentorial white matter

At the physical examination performed at age 21 months, the child displayed a set of clinical signs consisting of facial dysmorphic features, seizures, cognitive disability, and severe language delay. The episodes of epileptic seizures occurred frequently within short time intervals and were drug resistant. The weight was 8.5 kg, length 78 cm, and head circumference 43 cm (all below the <3rd percentile). Facial dysmorphism consisted of a small and receding forehead, sparse eyebrows, epicanthus, upslanting palpebral fissures, full cheeks, a wide nasal bridge, rounded nasal tip, large nares, a long phyltrum, small mouth, thin lips, a horizontal furrow under the lower lips, retrognathia, and a small nevus localized on the upper lip (Fig. 3 a–b). The ears were large and protruding with large lobules. The fingers were flexed with the hands partially closed, and the thumbs were long and adducted. The feet were long with a large big toe. In a recent examination at the age of 3 years, the weight was 11.2 kg (3rd percentile), length 85 cm (3rd percentile), and head circumference 43 cm (<3rd percentile) (Fig. 4) The child continued to show developmental delay and drug-resistant epileptic seizures. Moreover he presented daily febrile temperature ranging from 40 °C to 37 °C, without response to antipyretic drugs and without a clear infectious cause and without sweating. Nowaday, under anticonvulsivant treatment, the child showed a reduction of seizures, which appeared prevalently at awake and a series of two or three episodes at day. The daily thermal variation without a known cause led us to suppose as co-morbility, a thermal dysregulation of hypothalamic origin suggestive of reverse Shapiro’s syndrome. The MRI at the age of 3 years showed a moderate enlargement of both lateral ventricles and subarachnoid space of bilateral temporal pole with volume reduction of the supratentorial white matter (Fig. 5 a-b).

During the frequent admissions to the hospital, several specific analyses have been performed with the aim of defining the etiological event with the following results (normal values in parentheses): neopterin, 1.16 μg/L (2.30–10.10); biopterin, 2.88 μg/L (2.40–11.80); neurotransmitters 5-hydroxyindole acetic acid (5HIAA), 330 nmol/L (152–462); homovanillic acid (HVA), 268 nmol/L (302–845); 3–0-methyldopa, 36 nmol/L (< 100); 5-hydroxytryptophan (5-HTP), 7 nmol/L (> 10); 3-methoxy-4-hydroxyphenylglycol, 7 nmol/L (51–112); HVA/5HIAA 0.81 (1.50–3.50).

### Genetic testing

Genomic DNA isolation was carried out for the family trio (father, mother and child affected). Array Comparative Genome Hybridization (aCGH) was performed by CytoSure ISCA 8x60k array from Oxford Gene Technology (OGT), according to the manufacturer’s recommendations (Agilent Technologies, Santa Clara, CA). aCGH data were analyzed and interpreted using Cytosure software (GRCh38 assembly) provided by OGT. In addition to this, a targeting enrichment was performed using the Illumina TruSight One panel, a diagnostic panel of 47 EE genes containing probes to capture the exonic regions of 4813 genes associated with the clinical phenotype. The samples were sequenced by using the Illumina NextSeq 500 platform (Illumina Inc.) with 2 × 150 bp paired-end reads. Alignments and variant calls were generated using NextGene software (v2.4.1, 2015) and variant calls (with coverage <15X) were limited to the genes of interest. For the clinical interpretation of genomic variants was used Alamut-Batch (Version 1.4.0, 2015), the high-throughput annotation software for NGS analysis. Variants were annotated for minor allele frequencies in the Exome Aggregation Consortium (ExAC) database (Version 0.3), and heterozygous variants with minor allele frequencies > 0.01 (1%) were filtered out. Variants were classified as pathogenic/likely pathogenic/VOUS/likely benign/benign according to the 2015 American College of Medical Genetics and Genomics (ACMG) guidelines [17]. Alamut Visual (Version 2.7) was used for integrating genetic and predictive information on missense, nonsense, frameshift, and splice-site variants, providing computational algorithms for SIFT and PolyPhen-2 (Version 2.2.2, 2012). The validation of variants classified as pathogenic or likely pathogenic was performed using Sanger sequencing in the proband.

## Results

**Fig. 6:**
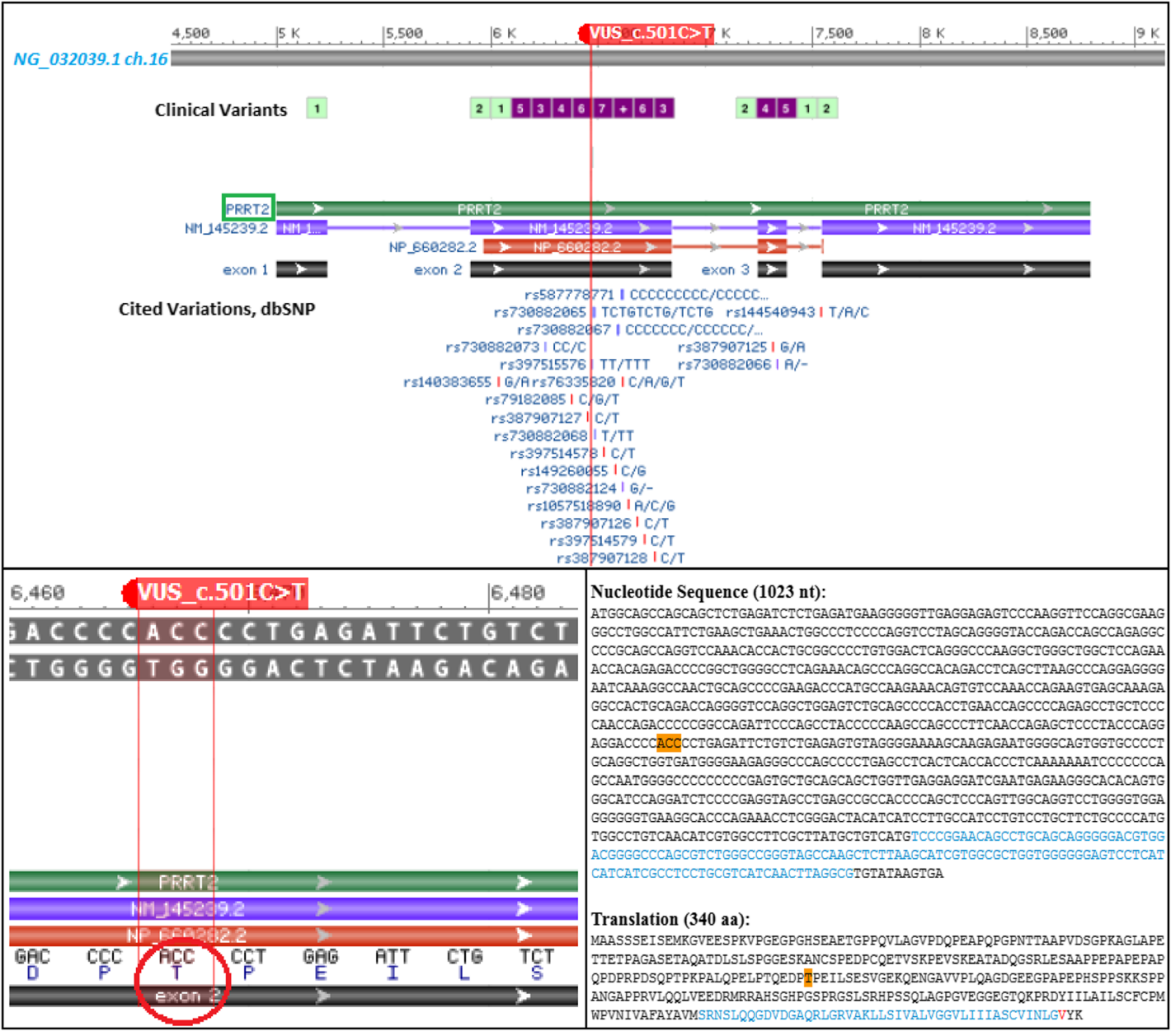
Schematic representation from NCBI Sequence Viewer 3.32.0 of the VUS detected by exome sequencing. The upper panel corresponds to the screenshot from NCBI Sequence Viewer showing the missense sequence variant c.501 C > T in the exon 2 of the *PRRT2* gene. The red line indicates the VUS position around which a range of clinical variants annotated in dbSNP flanking the site of mutation, thereby giving evidence of a region susceptible to gene expression variability. In the lower panel, on the left the red circle highlights the codon of interest, and on the right, it was shown the consensus coding sequence (CCDS) and the corresponding substitution of threonine 167 where occurs the mutated base

Molecular karyotype analysis did not show any copy number alterations. Using a targeted epileptic encephalopathy (EE) panel, we identified a heterozygous variant of uncertain significance (VUS) in the gene *PRRT2* (Fig. 6)*.* The variant results in a synonymous amino acid substitution (c.501 C > T; p.Thr 167 =) and was detected in the unaffected father, but not reported in the mother. The variant c.501 > C > T is likely benign (2015 ACMG classification) but, it is not observed in large population cohorts (gnomAD).

## Discussion

The boy presents with facial dysmorphism, congenital microcephaly, severe developmental delay, and drug-resistant epileptic encephalopathy. The main dysmorphic features consist of a small receding forehead, epicanthus, upslanting palpebral fissures, a large nasal bridge with a rounded tip, a long philtrum, small mouth, thin lips, horizontal furrow under the lower lip, and retrognathia. He was markedly hypotonic with severe cognitive disability. The epileptic seizures were initially of the early myoclonic type that subsequently transitioned to spasms in flexion associated with episodes of convulsive status epilepticus that required frequent admission to the intensive therapy unit. Serial EEG persisted in showing multifocal paroxysmal spike and wave activity. Brain MRI showed simplified cortical sulci and microcavitations, the latter possibly related to the severe hypoxic-ischemic encephalopathy (HIE) of perinatal origin. However, in this child the microcephaly was not linked to the HIE as it was prenatally recognized by uterine ultrasound and confirmed at birth by head circumference measurement less than the third percentile. In contrast to what has been observed with the present child, usually infants with HIE have a head circumference measurement within the normal range at birth. As reported in a study on 52 term infants, no statistical difference was noted between the head circumference of newborns with HIE and normal control infants [18]. The clinical manifestations of HIE have certainly played a role in the neurologic involvement of this child, but it must be considered a co-morbid event. The complex clinical features presented by the child, particularly the signs of severe epileptic encephalopathies led us to carry out a panel of molecular analyses related to these disorders, but the genes examined were not informative.

A diagnostic exome sequencing analysis of the proband detected a variant of uncertain significance for the *PRRT2* gene, which could have played a role in the clinical manifestations presented by the child. *PRRT2* encodes a protein expressed in the central nervous system that is mainly localized in the pre-synaptic neurons and involved in the modulation of synaptic neurotransmitter release. A reduction or no production of the *PRRT2* protein might cause alterations in synaptic neurotransmitter release and/or a dysregulation of the neuronal excitability in different sides of the brain, causing different types of neurological disorders [2, 19–22]. The *PRRT2* mutation has been predominantly related to individuals and families with a wide group of early onset paroxysmal disorders such as PKD and different types of benign infantile seizures [2, 4, 5]. A study on the incidence of *PRRT2* mutations in a cohort of 16 PKD patients and their relatives (a total of 39 individuals) was conducted by Lamperti et al. [3] that found *PRRT2* mutations in 10 of the 16 patients and in 23 relatives. In 27 of the 33 individuals the mutations was c.insC649 p.Arg217Profs*8. For this group of patients, the mean age of onset was 10 years, and the PKD episodes were generalized and ranged from a few minutes to several days. No associations with epileptic seizures or EEG abnormalities were reported. In another study of 11 patients with paroxysmal hypnogenic dyskinesia, mutations in the *PRRT2* gene were found in two typical patients [23]. In a cohort of Chinese families with members affected by paroxysmal kinesigenic dyskinesia, infantile convulsions, and choreoathetosis, direct sequencing showed three different pathogenic mutations (c.649dupC, c.776dupG, and c.649C → T) in the *PRRT2* gene [24]. These authors found no clear evidence of a genotype-phenotype correlation regarding the age of onset or the types of mutations [24]. Ebrahimi-Fakhari et al. [2] performed a wide review of 1444 published cases of *PRRT2* mutations using a systematic approach to the clinical and genetic characteristics of this group of patients. A positive family history was reported in 89.1% of the patients, and in 87.1% of the reported cases, the *PRRT2* mutations were familial in origin. In this study, benign familial infantile epilepsy was found in 602 infants (41.7%), paroxysmal kinesigenic dyskinesia in 560 (38.7%), and infantile convulsions and choreoathetosis in 206 (14.3%). Only 76 patients (i.e., 5.3%) presented with a primary diagnosis unrelated to these disorders.

As previously mentioned, the clinical spectrum of individuals affected by *PRRT2* gene mutations is wide, and it is difficult to correlate genotype with phenotype. The two more frequent clinical expressions of *PRRT2* mutations causing benign infantile epilepsies and paroxysmal dyskinesia tend to manifest with such different clinical expressions and ages of onset that it is difficult to provide a single etiological explanation for so different clinical manifestations [24]. The proband in our study presents unusual clinical features compared with those reported for other individuals affected with *PRRT2* mutations, and congenital microcephaly, severe brain involvement, and dysmorphisms have not been previously reported in patients with this mutation. *PRRT2* mutations were found in only a few cases when 70 genes were genetically tested in 8565 patients with epilepsy and neurodevelopmental disorders [16]. A report by Trump et al. [20] on a cohort of 400 patients with early onset epilepsy and severe developmental delay disorders found *PRRT2* mutations in only two patients. Delcourt et al. [14] support the hypothesis that homozygous or compound heterozygous deleterious *PRRT2* gene mutations may present with more severe clinical expression compared to those resulting from a single mutation [14]. In a study of 5 patients [14], the authors report complex phenotypes consisting of (1) a combination of at least three different forms of paroxysmal neurological disorders within the same patient and persistent paroxysmal attacks; (2) prolonged episodes of ataxia; and (3) associated neurologic disorders that include learning difficulties in four patients and cerebellar atrophy in two patients. Liu et al. [15] hypothesize that knocked down *PRRT2* expression in vivo might result in embryonic delay in neuronal migration with a marked reduction in synaptic density after birth.

We dealt with a complex clinical case for data interpretation. Gene panel sequencing failed to identify causal variants possibly correlated with the clinical signs presented by the child. The analysis did not show the presence of point mutations in the translated regions or in the intron-exon junctions of the examined EE genes. The missense variant in *PRRT2* gene (c.501C > T; p.Thr167Ile) reported in the proband and in the healthy father has not been associated as pathogenetic neither in the ClinVar database and Single Nucleotide Polymorphism Database (dbSNP) (Fig. 6, upper panel). Actually, this result does not exclude that the protein structural change has not proven sufficiently robust to be clinically useful.

Although the neutral mutation may have a minimal impact in the gene product, synonymous does not mean the same, therefore, an identical variation may impair the gene regulation and affect the site of conserved sequence motifs of transcription factors [25, 26]. It could not be excluded to also influence aberrant mRNA splicing and specific DNA methylation signatures [27–29].

**Fig. 7:**
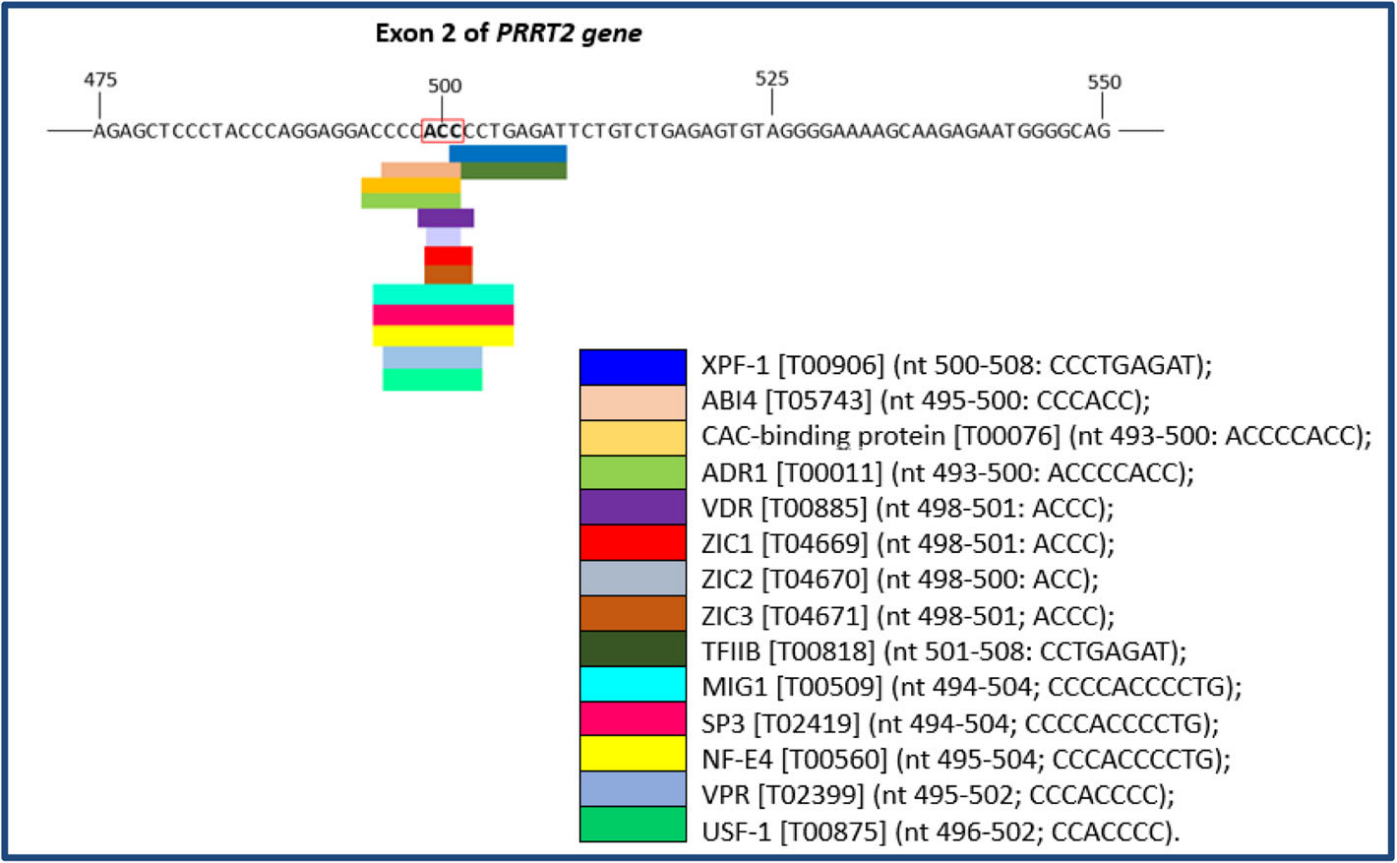
Analysis in silico of putative TFs using PROMO predicted for the coding consensus sequencing of *PRRT2* gene. Output modified from PROMO result showing the binding sites of TFs overlapping the variant c.501 C > T in the exon 2 of the PRRT2 gene

In attempt to evaluate the genomic context in which the VUS is included, we performed a predictive analysis for the identification of putative transcription factor binding sites (TFBS) in DNA sequence with the PROMO website tool (http://alggen.lsi.upc.es/cgi-bin/promo_v3/promo/promoinit.cgi?dirDB=TF_8.3), using data from TRANSFAC database version 8.3 [30, 31]. The PROMO output (Fig. 7) showed that the genomic position of VUS is enriched for 14 transcription factors (*XPF-1, ABI4, CAC-binding protein, ADR1, VDR, ZIC1, ZIC2, ZIC3, TFIIB, MIG1, SP3, NF-E4, VPR* and *USF-1*).

However, this report does not exclude that further studies on the conservation of the amino acid involved and conformational regulation in the downstream signaling protein interactions will be needed to investigate new insights into the pathogenicity of the non-synonymous substitutions. Intriguingly, according to all mutations reported to date, the exon 2 encoding the extracellular and the proline-rich domain of the protein is prevalently associated with a wide number of missense variants, some of those likely pathogenic, but the majority are likely benign, with intermediate predictions and without a specific disease setting [32]. Given the normal phenotype of the proband’s father, the role of the missense substitution may also underlie the complexity of a genetic-phenotype correlation and suggest the incomplete penetrance frequently encountered in dominantly inherited epilepsies. Furthermore, it may be hypothesized that in the affected child modifying factors may have acted in causing the severe clinical features. Genetic heterogeneity, the involvement of other genes or the non-coding regions of the *PRRT2* gene, as well as epigenetic effects may have acted during fetal development to provoke in the child severe clinical expressions.

In conclusion, the severe clinical features presented by the child in association with the missense variant of *PRRT2* might extend the spectrum of clinical manifestations [24]. We provided points to consider stimulating this debate across other similar observations, which could help to clarify the clinical interpretation of the VUS and other factors influencing the clinical expression of such a severe disorder [24, 33, 34].
